# Two new species of *Nanorana* Günther, 1896 (Anura, Dicroglossidae) from Yunnan, China

**DOI:** 10.3897/zookeys.1276.179415

**Published:** 2026-04-03

**Authors:** Shuo Liu, Chao Bu, Mian Hou, Jing Chen, Yanfei Feng, Lue Ma, JiShan Wang, Dingqi Rao

**Affiliations:** 1 Kunming Natural History Museum of Zoology, Kunming Institute of Zoology, Chinese Academy of Sciences, Kunming, Yunnan 650223, China Kunming Natural History Museum of Zoology, Kunming Institute of Zoology, Chinese Academy of Sciences Kunming China; 2 Yunnan Wenshan National Nature Reserve Management and Protection Bureau, Wenshan, Yunnan 663000, China Kunming Institute of Zoology, Chinese Academy of Sciences Kunming China; 3 College of Continuing (Online) Education, Sichuan Normal University, Chengdu, Sichuan 610066, China College of Continuing (Online) Education, Sichuan Normal University Chengdu China; 4 Yunnan Wumengshan National Nature Reserve Management and Protection Bureau, Zhaotong, Yunnan 657000, China Yunnan Wenshan National Nature Reserve Management and Protection Bureau Wenshan China; 5 Wenshan City Management and Protection Sub-Bureau of Yunnan Wenshan National Nature Reserve, Wenshan, Yunnan 663000, China Yunnan Wumengshan National Nature Reserve Management and Protection Bureau Zhaotong China; 6 Daguan County Forestry and Grassland Bureau, Daguan, Yunnan 652400, China Wenshan City Management and Protection Sub-Bureau of Yunnan Wenshan National Nature Reserve Wenshan China; 7 Southwest Survey and Planning Institute of National Forestry and Grassland Administration, Kunming, Yunnan 650031, China Daguan County Forestry and Grassland Bureau Daguan China; 8 Kunming Institute of Zoology, Chinese Academy of Sciences, Kunming, Yunnan 650201, China Southwest Survey and Planning Institute of National Forestry and Grassland Administration Kunming China

**Keywords:** Mitochondrion, morphology, phylogeny, taxonomy, Wenshan, Zhaotong

## Abstract

Two new species of the *Nanorana yunnanensis* complex are described from southeastern and northeastern Yunnan Province, China, respectively, based on morphological and molecular evidence. The two new species can be distinguished from the three known species of this complex and each other by the difference in the skin texture, the visibility of the tympanum, the ventral coloration, and the distribution of the spines on the ventral surface of the head in adult males. Phylogenetically, the two new species formed two distinct clades in the *N. yunnanensis* complex and differ from other species of the complex and each other by 2.4–5.0% in the 16S gene sequences and 9.8–11.3% in the ND2 gene sequences. This study brings the total number of recognized species of the genus *Nanorana* to 36, of which 27 occur in China and 13 in Yunnan.

## Introduction

*Nanorana* Günther, 1896, is a genus endemic to Asia in the family Dicroglossidae (Frost 2026). It is widely distributed from the Himalayan region and central China to northern Indochina (Ohler and Dubois 2006; Che et al. 2009, 2010; Li et al. 2010; Fei et al. 2012; Qi et al. 2019; Liu et al. 2021; Tang et al. 2023; Hofmann et al. 2024; Frost 2026). The genus currently contains 34 species, 25 of which were recorded in China (Liu et al. 2025; AmphibiaChina 2026; Frost 2026).

Among the genus, the *Nanorana yunnanensis* complex is a group of larger sized frogs with two patches of chest spines in males, no dorsolateral folds, and usually no obvious patterns on dorsum (Fei et al. 2005, 2009). Due to the extreme morphological similarities and the lack of phylogenetic research in early taxonomic studies, species of this complex are often misidentified. This complex has undergone multiple taxonomic changes and has involved five species, namely *Rana yunnanensis* Anderson, 1879, *Rana phrynoides* Boulenger, 1917, *Rana sichuanensis* Dubois, 1987, *Rana liui* Dubois, 1987, and *Rana bourreti* Dubois, 1987 (Huang et al. 2016). Zhang et al. (2010) conducted phylogenetic and phylogeographic analysis on a large number of samples of the *N. yunnanensis* complex and found that they formed three highly divergent lineages, but they did not make a taxonomic decision. Huang et al. (2016) reviewed the taxonomic history of this complex and reassessed it through extensive sampling and morphological and phylogenetic analysis and demonstrated three species in the *N. yunnanensis* complex, namely *N. phrynoides* (Boulenger, 1917), *N. sichuanensis* (Dubois, 1987), and *N. yunnanensis* (Anderson, 1879), with *R. liui* being a synonym of *N. sichuanensis* and *R. bourreti* being a synonym of *N. yunnanensis*. Currently, AmphibiaChina (2026) adopts the viewpoint of Huang et al. (2016) and recognizes the three names *N. phrynoides*, *N. sichuanensis*, and *N. yunnanensis*. However, Frost (2026) considered that the name *N. yunnanensis* actually applies to what was previously referred to as *N. phrynoides* since the neotype (BMNH 1947.2.3.76) of *N. yunnanensis* is the lectotype of *N. phrynoides*, and the oldest name for the previous *N. yunnanensis* should be referred to as *N. bourreti* (Dubois, 1987). Therefore, the website Amphibian Species of the World currently listed *N. bourreti*, *N. sichuanensis*, and *N. yunnanensis* as valid species, and treated *R. phrynoides* as a synonym of *N. yunnanensis* (Frost 2026).

When we were sorting the specimens collected in the past, we found some specimens identified as *Nanorana phrynoides* and *N. sichuanensis* which were collected from Wenshan and Zhaotong cities, Yunnan Province, China, respectively. However, detailed morphological and molecular analyses indicated that these specimens do not belong to any known species, but two unnamed species in the *N. yunnanensis* complex. Herein, we describe and illustrate them as two new species.

## Materials and methods

All specimens new to this study have been deposited at Kunming Natural History Museum of Zoology, Kunming Institute of Zoology, Chinese Academy of Sciences (**KIZ**). Genomic DNA was extracted from liver or muscle tissues excised from the specimens. The mitochondrion 16S ribosomal RNA gene (16S) and NADH dehydrogenase subunit 2 gene (ND2) were amplified and sequenced. Primers used for 16S were L2188 (5’-AAAGTGGGCCTAAAAGCAGCCA-3’) and 16H1 (5’-CTCCGGTCTGAACTCAGATCACGTAGG-3’) (Hedges 1994; Matsui et al. 2006) and for ND2 were MTH (5’-AAGCTTTTGGGCTCATGCC-3’) and Htrp (5’-TGCTGAGAACTTTGAAGG-3’) (Huang et al. 2016). The molecular experiment was completed by Sangon Biotech (Shanghai, China) Co., Ltd. *Nanorana unculuanus* (Liu, Hu & Yang, 1960), *N. quadranus* (Liu, Hu & Yang, 1960), and *N. pleskei* Günther, 1896 were used as out groups of the *N. yunnanensis* complex according to Liu et al. (2021, 2025). The sequences of congeners and out groups were obtained from GenBank (Table 1).

**Table 1.** Samples used in the phylogenetic analysis of this study.

Taxon	Voucher	16S	ND2	Reference
Clade A	CIB20060609	/	KU140125	Huang et al. 2016
Clade A	CIB20060610	/	KU140126	Huang et al. 2016
Clade A	CIB20060611	/	KU140127	Huang et al. 2016
Clade A	CIB20060612	/	KU140128	Huang et al. 2016
Clade A	CIB20060276	KU140033	KU140129	Huang et al. 2016
Clade A	CIB20060616	KU140035	KU140130	Huang et al. 2016
Clade A	CIB20060607	KU140034	KU140131	Huang et al. 2016
Clade A	CIB20070433	KU140030	KU140132	Huang et al. 2016
Clade A	CIB20070437	KU140031	KU140133	Huang et al. 2016
Clade A	CIB20070434	/	KU140134	Huang et al. 2016
Clade A	CIB20070436	KU140032	KU140135	Huang et al. 2016
Clade A	CIB20060570	KU140037	KU140136	Huang et al. 2016
Clade A	CIB20070431	/	KU140137	Huang et al. 2016
Clade A	CIB20070432	/	KU140138	Huang et al. 2016
Clade A	CIB20070435	/	KU140139	Huang et al. 2016
Clade A	CIB20060592	KU140038	KU140140	Huang et al. 2016
Clade A	CIB20060589	KU140036	KU140141	Huang et al. 2016
Clade A	CIB20060593	/	KU140142	Huang et al. 2016
Clade A	CIB20050016	/	KU140143	Huang et al. 2016
Clade A	CIB200049	KU140039	KU140144	Huang et al. 2016
Clade A	CIB200046	/	KU140145	Huang et al. 2016
Clade A	CIB200044	KU140040	KU140146	Huang et al. 2016
Clade A	CIBYN090022	/	KU140147	Huang et al. 2016
Clade A	CIBYN090023	KU140041	KU140148	Huang et al. 2016
Clade A	CIBYN090025	KU140042	KU140149	Huang et al. 2016
Clade A	CIBYN090026	/	KU140150	Huang et al. 2016
Clade A	CIBYN090016	/	KU140151	Huang et al. 2016
Clade A	CIBYN090017	KU140043	KU140152	Huang et al. 2016
Clade A	CIBYN090024	/	KU140153	Huang et al. 2016
Clade A	CIBYN090021	/	KU140154	Huang et al. 2016
Clade A	CIBYN090027	/	KU140155	Huang et al. 2016
Clade A	CIBYN090019	/	KU140156	Huang et al. 2016
Clade A	CIBYN090020	/	KU140157	Huang et al. 2016
Clade A	CIBYN090185	KU140044	KU140158	Huang et al. 2016
Clade A	CIBYN090186	/	KU140159	Huang et al. 2016
Clade A	PZH200806108	/	KU140160	Huang et al. 2016
Clade A	CIB20080193	/	KU140161	Huang et al. 2016
Clade A	PZH200806109	/	KU140162	Huang et al. 2016
Clade A	PZH200806115	/	KU140163	Huang et al. 2016
Clade A	CIB20080187	/	KU140164	Huang et al. 2016
Clade A	CIB20080191	/	KU140165	Huang et al. 2016
Clade A	CIB20080190	/	KU140166	Huang et al. 2016
Clade A	CIB20080189	/	KU140167	Huang et al. 2016
Clade A	CIBYN2008060601	KU140047	KU140168	Huang et al. 2016
Clade A	CIBYN2008061002	KU140048	KU140169	Huang et al. 2016
Clade A	CIBYN2008061003	KU140049	KU140170	Huang et al. 2016
Clade A	CIBXM2925	KU140045	KU140171	Huang et al. 2016
Clade A	CIBXM2927	/	KU140172	Huang et al. 2016
Clade A	CIBPZH200806108	KU140046	/	Huang et al. 2016
Clade A	CIBYN2008061001	/	KU140173	Huang et al. 2016
Clade A	CIB20070428	/	KU140174	Huang et al. 2016
Clade A	CIB20070430	/	KU140175	Huang et al. 2016
Clade A	CIB20070426	/	KU140176	Huang et al. 2016
Clade A	CIB20070454	/	KU140177	Huang et al. 2016
Clade A	CIB20070429	/	KU140178	Huang et al. 2016
Clade A	CIB2968K	/	KU140179	Huang et al. 2016
Clade A	CIB20070427	/	KU140180	Huang et al. 2016
Clade A	CIB2959K	KU140061	KU140181	Huang et al. 2016
Clade A	CIBYY20080656	KU140062	KU140182	Huang et al. 2016
Clade A	CIBYY20080654	/	KU140183	Huang et al. 2016
Clade A	CIBYY20080661	/	KU140184	Huang et al. 2016
Clade A	CIBYY20080669	/	KU140185	Huang et al. 2016
Clade A	CIBYY20080658	/	KU140186	Huang et al. 2016
Clade A	CIBYY20080655	/	KU140187	Huang et al. 2016
Clade A	CIBYY20080663	/	KU140188	Huang et al. 2016
Clade A	CIBYY20080667	/	KU140189	Huang et al. 2016
Clade A	CIBYY20080668	/	KU140190	Huang et al. 2016
Clade A	CIBYY20080659	/	KU140191	Huang et al. 2016
Clade A	CIBLGH20080638	/	KU140192	Huang et al. 2016
Clade A	CIBLGH20080676	/	KU140193	Huang et al. 2016
Clade A	CIBLGH20080678	/	KU140194	Huang et al. 2016
Clade A	CIBLGH20080675	/	KU140195	Huang et al. 2016
Clade A	CIBLGH20080673	/	KU140196	Huang et al. 2016
Clade A	CIBLGH20080674	/	KU140197	Huang et al. 2016
Clade A	CIBLGH20080639	/	KU140198	Huang et al. 2016
Clade A	CIBLGH20080677	/	KU140199	Huang et al. 2016
Clade A	CIBLGH20080672	KU140060	KU140200	Huang et al. 2016
Clade A	CIBLGH20080637	KU140059	KU140201	Huang et al. 2016
Clade A	CIBYY20080679	KU140063	KU140202	Huang et al. 2016
Clade A	CIBYY20080693	KU140064	KU140203	Huang et al. 2016
Clade A	CIBYY20080680	/	KU140204	Huang et al. 2016
Clade A	CIBYY20080630	/	KU140205	Huang et al. 2016
Clade A	CIBYY20080626	/	KU140206	Huang et al. 2016
Clade A	CIBYY20080683	/	KU140207	Huang et al. 2016
Clade A	CIBYY20080682	/	KU140208	Huang et al. 2016
Clade A	CIBYY20080681	/	KU140209	Huang et al. 2016
Clade A	CIBYY20080684	/	KU140210	Huang et al. 2016
Clade A	CIB20070722003	KU140050	KU140211	Huang et al. 2016
Clade A	CIB20070722002	KU140051	KU140212	Huang et al. 2016
Clade A	CIB20060435	KU140053	KU140213	Huang et al. 2016
Clade A	CIB20060436	KU140052	KU140214	Huang et al. 2016
Clade A	CIBYN2008060214	KU140055	KU140215	Huang et al. 2016
Clade A	CIBYN2008060201	KU140054	KU140216	Huang et al. 2016
Clade A	CIBYN2008060202	/	KU140217	Huang et al. 2016
Clade A	CIBYN2008060207	/	KU140218	Huang et al. 2016
Clade A	CIBYN2008060209	/	KU140219	Huang et al. 2016
Clade A	CIBYN2008060205	/	KU140220	Huang et al. 2016
Clade A	CIBYN2008060208	/	KU140221	Huang et al. 2016
Clade A	CIBYN2008060203	/	KU140222	Huang et al. 2016
Clade A	CIBYN2008060206	/	KU140223	Huang et al. 2016
Clade A	CIBYN2008060212	KU140058	KU140224	Huang et al. 2016
Clade A	CIBYN2008060413	/	KU140225	Huang et al. 2016
Clade A	CIBYN2008060402	/	KU140226	Huang et al. 2016
Clade A	CIBYN2008060403	/	KU140227	Huang et al. 2016
Clade A	CIBYN2008060404	/	KU140228	Huang et al. 2016
Clade A	CIBYN2008060407	/	KU140229	Huang et al. 2016
Clade A	CIBYN2008060414	/	KU140230	Huang et al. 2016
Clade A	CIBYN2008060406	/	KU140231	Huang et al. 2016
Clade A	CIBYN2008060401	KU140056	KU140232	Huang et al. 2016
Clade A	CIBYN2008060415	KU140057	KU140233	Huang et al. 2016
Clade A	CIBYN2008052901	KU140027	KU140234	Huang et al. 2016
Clade A	CIBYN2008053007	/	KU140235	Huang et al. 2016
Clade A	CIBYN2008053013	KU140028	KU140236	Huang et al. 2016
Clade A	CIBYN2008053008	KU140029	KU140237	Huang et al. 2016
Clade A	CIBYN2008053006	/	KU140238	Huang et al. 2016
Clade A	CIBYN2008053012	/	KU140239	Huang et al. 2016
Clade A	CIBYN2008053014	/	KU140240	Huang et al. 2016
Clade A	CIBYN2008053010	/	KU140241	Huang et al. 2016
Clade A	CIBYN2008053009	/	KU140242	Huang et al. 2016
Clade A	CIBYN2008053005	/	KU140243	Huang et al. 2016
Clade A	CIB20060569	GQ225873	GQ225877	Wang et al. 2009
Clade A	CIBJF003	GQ225874	GQ225878	Wang et al. 2009
Clade A	SCUM045183CJ	DQ118492	/	Che et al. 2009
Clade A	YNU-HU2004009	DQ118494	/	Che et al. 2009
Clade A	YNU-HU200208005	EU979823	/	Che et al. 2009
Clade A	SCUM20030091GP	EU979824	/	Che et al. 2009
Clade A	YNU-HU20024012	EU979825	/	Che et al. 2009
Clade A	KIZ2025079	PZ096902	/	This study
Clade A	KIZ2025080	PZ096903	/	This study
Clade A	KIZ2025082	PZ096904	/	This study
Clade A	KIZ2025084	PZ096905	/	This study
Clade A	KIZ2025085	PZ096906	/	This study
Clade A	KIZ2025086	PZ096907	/	This study
Clade A	KIZ2025087	PZ096908	/	This study
Clade A	KIZ2025092	PZ096909	/	This study
Clade A	KIZ2025095	PZ096910	/	This study
Clade A	KIZ2025096	PZ096911	/	This study
Clade B	CIBYN090608	KU140003	KU140088	Huang et al. 2016
Clade B	CIBYN090609	KU140004	/	Huang et al. 2016
Clade B	CIBYN090610	KU140005	/	Huang et al. 2016
Clade B	CIB980064	KU140006	/	Huang et al. 2016
Clade B	CIBYN09060302	KU140009	KU140089	Huang et al. 2016
Clade B	CIBYN09060102B	/	KU140090	Huang et al. 2016
Clade B	CIBYN09060103B	/	KU140091	Huang et al. 2016
Clade B	CIBYN09060101B	KU140008	KU140092	Huang et al. 2016
Clade B	CIBYN09060105B	/	KU140093	Huang et al. 2016
Clade B	CIBYN09060106B	/	KU140094	Huang et al. 2016
Clade B	CIBYN09060201B	KU140012	KU140095	Huang et al. 2016
Clade B	CIBYN09060203B	KU140013	KU140096	Huang et al. 2016
Clade B	CIBYN09060204B	/	KU140097	Huang et al. 2016
Clade B	CIBYN09060205B	KU140014	KU140098	Huang et al. 2016
Clade B	CIB20070708007	/	KU140099	Huang et al. 2016
Clade B	CIBXM3163	/	KU140100	Huang et al. 2016
Clade B	CIB20070709003	/	KU140101	Huang et al. 2016
Clade B	CIB20070709002	KU140015	KU140102	Huang et al. 2016
Clade B	CIB20070711007	/	KU140103	Huang et al. 2016
Clade B	CIB20070711005	/	KU140104	Huang et al. 2016
Clade B	CIB20070711004	/	KU140105	Huang et al. 2016
Clade B	CIBYN090181	/	KU140106	Huang et al. 2016
Clade B	CIBYN090035	KU140024	KU140107	Huang et al. 2016
Clade B	CIBYN090037	/	KU140108	Huang et al. 2016
Clade B	CIBYN090038	KU140026	KU140109	Huang et al. 2016
Clade B	CIBYN090069	KU140021	KU140110	Huang et al. 2016
Clade B	CIBYN090071	KU140023	KU140111	Huang et al. 2016
Clade B	CIBYN090072	/	KU140112	Huang et al. 2016
Clade B	CIB20090047	KU140019	KU140113	Huang et al. 2016
Clade B	CIB20090046	KU140018	KU140114	Huang et al. 2016
Clade B	CIBYN09060104B	/	KU140115	Huang et al. 2016
Clade B	CIBYN090070	KU140022	KU140116	Huang et al. 2016
Clade B	CIBYN090036	KU140025	KU140117	Huang et al. 2016
Clade B	CIB20070711006	/	KU140118	Huang et al. 2016
Clade B	CIB20070711009	KU140016	KU140119	Huang et al. 2016
Clade B	CIB20090045	KU140017	/	Huang et al. 2016
Clade B	CIB20090059	KU140020	/	Huang et al. 2016
Clade B	CIBXM2965	/	KU140120	Huang et al. 2016
Clade B	CIBYN09060202B	/	KU140121	Huang et al. 2016
Clade B	CIBYN09060304	KU140010	KU140122	Huang et al. 2016
Clade B	CIBYN09060305	KU140011	KU140123	Huang et al. 2016
Clade B	YNU-HU20010505	DQ118495	/	Che et al. 2009
Clade B	ROM27866	EU979826	/	Che et al. 2009
Clade B	ROM41507	EU979827	/	Che et al. 2009
Clade B	YNU-HU20010401	EU979828	/	Che et al. 2009
Clade B	YNU-HU20011102	EU979829	/	Che et al. 2009
Clade B	ROM19128	AF206470	/	Chen et al. 2005
Clade B	KIZ047053	KU599876	/	Suwannapoom et al. 2016
Clade B	KIZ2025088	PZ096912	/	This study
Clade B	KIZ2025089	PZ096913	/	This study
Clade B	KIZ2025090	PZ096914	/	This study
Clade C	CIBYN2008053001	KU139986	KU140065	Huang et al. 2016
Clade C	CIBYN2008053002	KU139987	KU140066	Huang et al. 2016
Clade C	CIBYN2008053004	KU139989	KU140067	Huang et al. 2016
Clade C	CIBYN2008053003	KU139988	KU140068	Huang et al. 2016
Clade C	CIB20070369	KU139993	KU140069	Huang et al. 2016
Clade C	CIB20080181	KU139994	KU140070	Huang et al. 2016
Clade C	CIBYN2008061206	/	KU140071	Huang et al. 2016
Clade C	CIBYN2008061204	/	KU140072	Huang et al. 2016
Clade C	CIBYN2008061207	/	KU140073	Huang et al. 2016
Clade C	CIBYN2008061205	/	KU140074	Huang et al. 2016
Clade C	CIB20080179	KU139996	KU140075	Huang et al. 2016
Clade C	CIB20070370	KU139992	KU140076	Huang et al. 2016
Clade C	CIB20080178	KU139991	KU140077	Huang et al. 2016
Clade C	CIB20080180	KU139995	KU140078	Huang et al. 2016
Clade C	WG20080723001	KU139997	KU140079	Huang et al. 2016
Clade C	WG20080723002	KU139998	KU140080	Huang et al. 2016
Clade C	WG20080723003	KU139999	KU140081	Huang et al. 2016
Clade C	CIBYN090212	KU140000	/	Huang et al. 2016
Clade C	CIB20080318	KU139990	KU140082	Huang et al. 2016
Clade C	CIBYN090222	KU140001	KU140083	Huang et al. 2016
Clade C	CIBYN090224	/	KU140085	Huang et al. 2016
Clade C	CIBYN090225	/	KU140086	Huang et al. 2016
Clade C	CIBYN090251	/	KU140087	Huang et al. 2016
Clade C	STJW-LP-001	KF199150	KF199150	Zhang et al. 2018
Clade C	KIZ040249	PZ096915	/	This study
Clade C	KIZ040250	PZ096916	/	This study
Clade C	KIZ040251	PZ096917	/	This study
Clade C	KIZ040252	PZ096918	/	This study
Clade C	KIZ040253	PZ096919	/	This study
Clade C	KIZ030082	PZ096920	/	This study
Clade C	KIZ020999	PZ096921	/	This study
*Nanorana wenshanensis* sp. nov.	KIZ2015011	PZ096922	PZ096024	This study
*Nanorana wenshanensis* sp. nov.	KIZ2015051	PZ096923	PZ096025	This study
*Nanorana wenshanensis* sp. nov.	KIZ2015052	PZ096924	PZ096026	This study
*Nanorana wenshanensis* sp. nov.	KIZ2015053	PZ096925	PZ096027	This study
*Nanorana wenshanensis* sp. nov.	KIZ2015054	PZ096926	PZ096028	This study
*Nanorana wenshanensis* sp. nov.	KIZ2015055	PZ096927	PZ096029	This study
*Nanorana wenshanensis* sp. nov.	KIZ2015056	PZ096928	PZ096030	This study
*Nanorana wenshanensis* sp. nov.	KIZ2015057	PZ096929	PZ096031	This study
*Nanorana zhaotongensis* sp. nov.	KIZ2015097	PZ096930	PZ096032	This study
*Nanorana zhaotongensis* sp. nov.	KIZ2015098	PZ096931	PZ096033	This study
*Nanorana zhaotongensis* sp. nov.	KIZ2015114	PZ096932	PZ096034	This study
*Nanorana zhaotongensis* sp. nov.	KIZ2015115	PZ096933	PZ096035	This study
*Nanorana zhaotongensis* sp. nov.	KIZ2015116	PZ096934	PZ096036	This study
*Nanorana zhaotongensis* sp. nov.	KIZ2015117	PZ096935	PZ096037	This study
*Nanorana zhaotongensis* sp. nov.	KIZ2015118	PZ096936	PZ096038	This study
*Nanorana pleskei*	CIBF97034	GQ225875	GQ225879	Wang et al. 2009
*Nanorana quadranus*	CIB20060533	GQ225910	GQ225936	Wang et al. 2009
*Nanorana unculuanus*	YNU-HU2002502702	DQ118491	HM054414	Che et al. 2009; Zhang et al. 2010

DNA sequences were aligned using MAFFT 7 (Katoh and Standley 2013). The uncorrected pairwise distances between species were calculated in MEGA 11.0.13 (Tamura et al. 2021). Bayesian inference and maximum likelihood analysis were used to construct the phylogenetic tree using the concatenated 16S and ND2 fragments. The best substitution models were selected using the corrected Akaike information criterion in ModelFinder (Kalyaanamoorthy et al. 2017). Due to the varying rates of evolution of different gene segments and different codons of encoding genes, the substitution models were selected separately for different gene segments and different codons of encoding genes (Kimura 1980; Arenas 2015). Bayesian phylogenetic inference was performed in MrBayes 3.2.6 (Ronquist et al. 2012) based on the GTR+F+G4 model for the first and third codon positions of ND2 and the GTR+F+I+G4 model for the second codon position of ND2 and 16S. The Markov chains were run for 10,000,000 generations and sampled every 100 generations. Maximum likelihood analysis was performed in IQ-TREE 1.6.12 (Nguyen et al. 2015) based on the K3Pu+F+G4 model for the first codon position of ND2, the TPM3+F+R2 model for the second codon position of ND2, the GTR+F+R2 model for the third codon position of ND2, and the TIM2+F+I+G4 model for 16S. The nodal support was estimated by 1,000 ultrafast bootstrap replicates.

The morphological measurements were taken with digital calipers to the nearest 0.1 mm. Measurement methods followed Huang et al. (2016). The morphometrics and character terminology include:

**AED** distance between anterior corners of eyes

**ED** diameter of eye

**FL** foot length

**HaL** hand length

**HL** head length

**HW** head width

**IML** length of inner metatarsal tubercle

**IMW** width of inner metatarsal tubercle

**INS** internasal space

**IOS** interorbital space

**IPTL** length of inner metacarpal tubercle

**IPTW** width of inner metacarpal tubercle

**LAHL** length of lower arm and hand

**NED** distance between nostril and eye

**NSD** distance between nostril and snout

**OPTL** length of outer metacarpal tubercle

**OPTW** length of outer metacarpal tubercle

**PED** distance between posterior corners of eyes

**SL** snout length

**SVL** snout-vent length

**TD** diameter of tympanum

**TED** distance from anterior edge of tympanum to posterior corner of eye

**TFL** length of foot and tarsus

**TiL** thigh length

**TL** tibia length

**TW** tibia width

**UEW** width of upper eyelid

In addition to the specimens of the two potential new species, we also examined 135 specimens of the three known species of the *Nanorana yunnanensis* complex. Principal component analysis was used to test whether there are morphological differences between species of the complex. Male and female were analyzed separately. Principal component analysis was performed using the prcomp command in R 4.2.2, and the scatter plot was plotted using the ggplot2 package in R 4.2.2 (R Core Team 2022).

## Results

The alignment of the 16S sequences contained 868 nucleotides, of which 739 sites were conserved and 129 sites were variable, including 96 parsimony-informative sites. The alignment of the ND2 sequences contained 1,032 nucleotides, of which 605 sites were conserved and 427 sites were variable, including 318 parsimony-informative sites.

**Figure 1.**
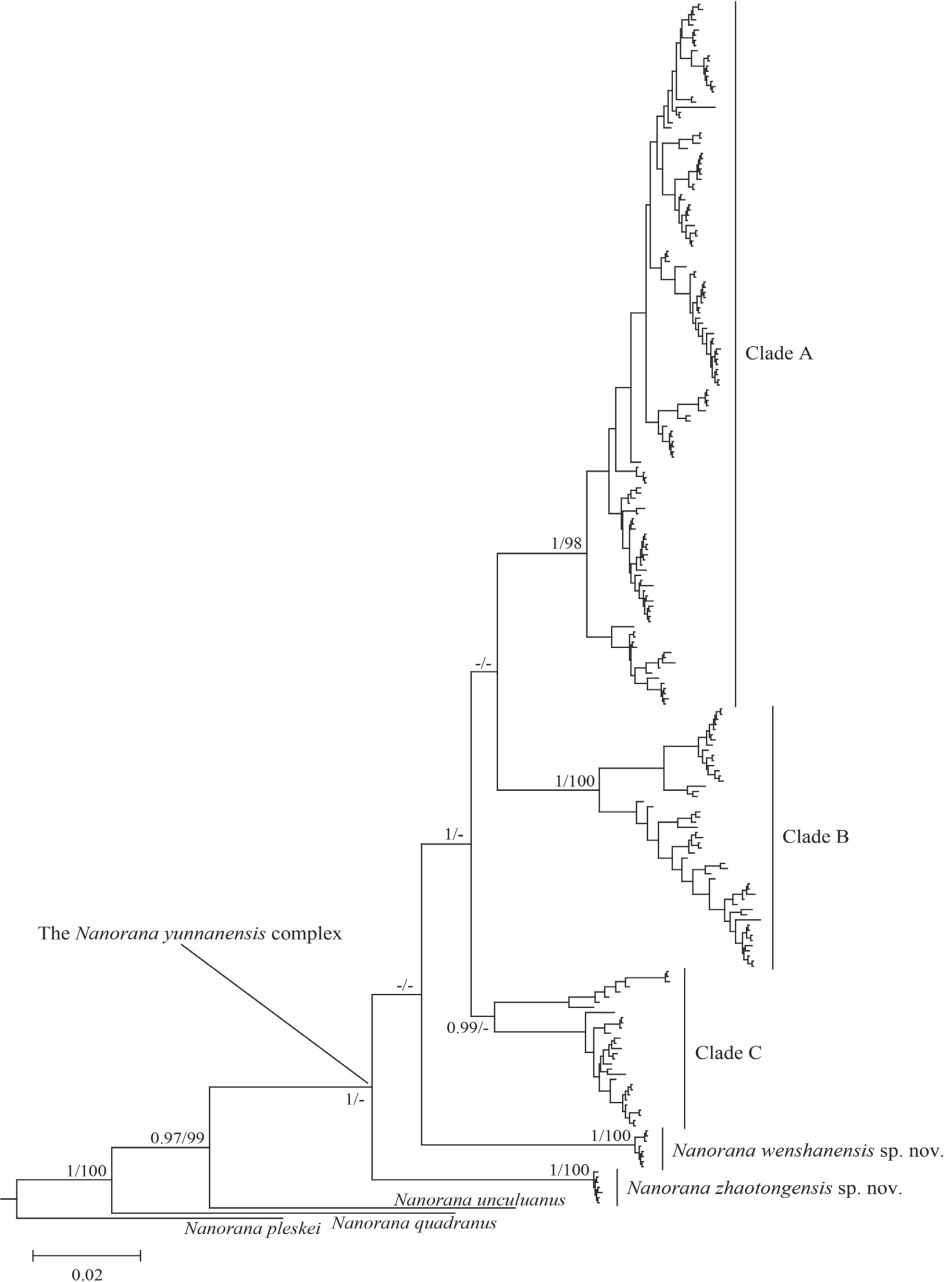
Bayesian phylogeny tree of the *Nanorana yunnanensis* complex based on concatenated 16S and ND2 sequences. Node numbers indicate Bayesian posterior probabilities/ultrafast bootstrap support for maximum likelihood analyses, “-” indicates values below 0.90 in Bayesian posterior probabilities or below 90 in bootstrap support for maximum likelihood analyses.

The results from Bayesian inference and maximum likelihood phylogenetic analysis are similar. The *Nanorana yunnanensis* complex was recovered into five highly divergent clades, of which clades A to C represent the three known species, while the other two new clades represent two previously unnamed species (Fig. 1).

The uncorrected pairwise distances between the two new clades and clades A to C ranged from 2.4% to 3.4% and the uncorrected pairwise distance between the two new clades was 5.0% calculated from the 16S sequences (Table 2).

**Table 2.** Uncorrected p-distances (%) calculated from 16S gene sequences.

	1	2	3	4	5	6	7
1 Clade A							
2 Clade B	2.2						
3 Clade C	2.7	2.0					
4 *Nanorana wenshanensis* sp. nov.	2.4	2.9	2.4				
5 *Nanorana zhaotongensis* sp. nov.	3.4	3.2	3.3	5.0			
6 *Nanorana pleskei*	4.1	4.5	4.3	4.1	5.2		
7 *Nanorana quadranus*	4.9	4.9	5.4	5.0	5.4	5.2	
8 *Nanorana unculuanus*	5.8	5.9	5.3	5.6	6.0	5.0	6.6

The uncorrected pairwise distances between the two new clades and clades A to C ranged from 9.8% to 11.3% and the uncorrected pairwise distance between the two new clades was 11.2% calculated from the ND2 sequences (Table 3).

**Table 3.** Uncorrected p-distances (%) calculated from ND2 gene sequences.

	1	2	3	4	5	6	7
1 Clade A							
2 Clade B	8.5						
3 Clade C	7.7	8.6					
4 *Nanorana wenshanensis* sp. nov.	10.0	10.2	9.8				
5 *Nanorana zhaotongensis* sp. nov.	11.3	10.8	10.5	11.2			
6 *Nanorana pleskei*	18.0	17.8	18.2	18.6	16.8		
7 *Nanorana quadranus*	17.2	17.5	17.5	16.1	17.5	17.4	
8 *Nanorana unculuanus*	15.4	15.7	15.9	15.3	15.0	17.5	15.8

**Figure 2.**
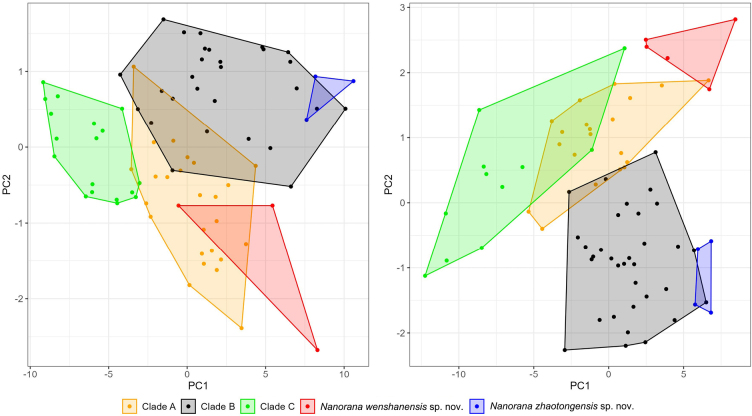
Scatterplot of the first two major principal components based on 27 morphometric characteristics (SVL, HL, HW, SL, ED, UEW, IOS, INS, NSD, NED, AED, PED, TD, TED, LAHL, HaL, IPTL, IPTW, OPTL, OPTW, TiL, TL, TW, TFL, FL, IML, and IMW) for males (left) and females (right) of the *Nanorana yunnanensis* complex.

The information of the examined specimens and the detailed measurement data can be found in Appendix 1 and Suppl. material 1. The first two major principal components from principal component analysis were retained, which accounted for 86.46% and 79.56% of the total variances for male and female, respectively. The clusters of the five clades each occupied a position in the morphospace with some overlap between others (Fig. 2). This indicates that there is relatively large intraspecific variation, and different species cannot be stably distinguished based on metric characteristics.

Although it is difficult to distinguish different clades based on measurement data, there are relatively significant differences in other morphological characteristics between these clades, such as skin texture, the visibility of the tympanum, and the ventral coloration. Combining the results of the phylogenetic analysis, we identify the two new clades as two new species and we formally describe them below.

### Taxonomic accounts

#### Nanorana wenshanensis sp. nov.

Taxon classificationAnimaliaAnuraDicroglossidae

F5EC739D-7BF3-5D4C-8E5D-85BCD32F08FE

https://zoobank.org/042A2961-1F8A-41ED-81C7-B04680DB9A1B

Figs 3, 4, 5

##### Type material.

***Holotype***. • KIZ2015051, adult male, collected on 16 April 2015 by Chao Bu from Bozhu Town, Wenshan City, Wenshan Prefecture, Yunnan Province, China (23°22'4"N, 103°52'0"E, 2180 m asl). ***Paratypes***. • KIZ2015052 and KIZ2015011, two adult males; • KIZ2015053–KIZ2015057, five adult females, all the same collection information as the holotype.

**Figure 3.**
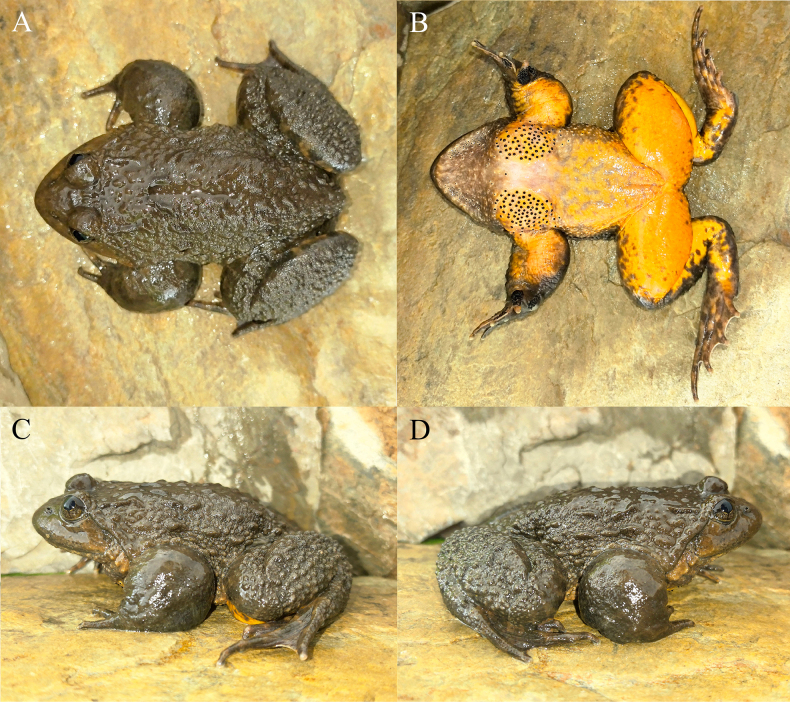
The holotype (KIZ2015051) of *Nanorana wenshanensis* sp. nov. in life. **A**. Dorsal view; **B**. Ventral view; **C**. Left view; **D**. Right view.

**Figure 4.**
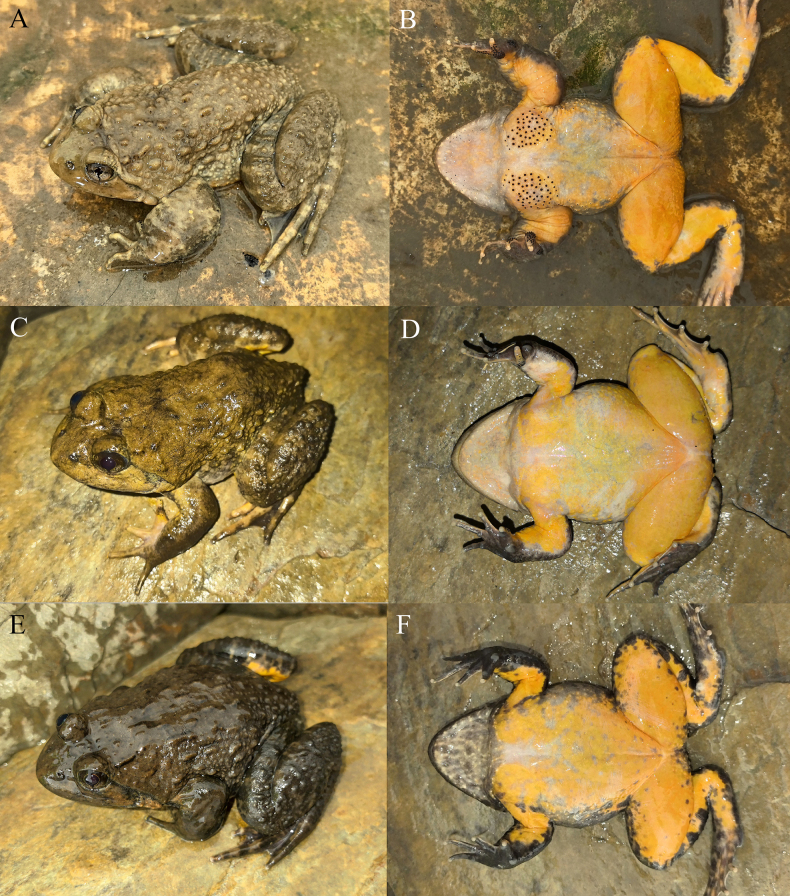
The paratypes of *Nanorana wenshanensis* sp. nov. in life. **A, B**. The male KIZ2015011; **C, D**. The female KIZ2015055; **E, F**. The female KIZ2015056.

**Figure 5.**
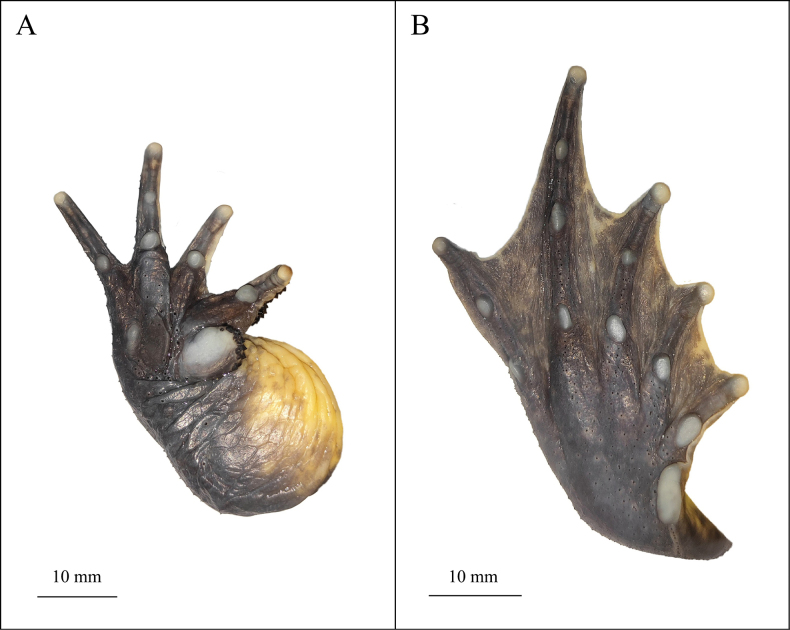
Close-up views of the hand and foot of the holotype (KIZ2015051) of *Nanorana wenshanensis* sp. nov. in preservative. **A**. Volar view of the right hand; **B**. Plantar view of the right foot.

##### Diagnosis.

Large sized spiny frog, SVL 83–107 mm in adult males and 94–110 mm in adult females; dorsal skin rough with many rounded, oval, and elongated warty tubercles; tympanum indistinct; finger I slightly longer than finger II; toe III slightly longer than toe V; toes fully webbed but obviously incurved between toes; tarsal fold present; dorsal surface light brown to dark brown; dark transversal bands on limbs quite indistinct; forelimbs strongly hypertrophied, keratinized spines present on chest, inner metacarpal tubercle, and fingers I to III, and internal vocal sac present in adult males.

##### Description of holotype.

Adult male, habitus stout, SVL 102.3 mm; head broader than long (HW/HL 1.13); snout relatively short (SL/HL 0.42), blunt and rounded in dorsal and lateral views; canthus rostralis indistinct; tympanum visible but indistinct; supratympanic fold distinct, from posterior corner of eye to before forelimb insertion; eye moderate (ED/HL 0.25), pupil almost oval; choanae oval; vomerine teeth distinct; tongue pear shaped, notched posteriorly; vocal sac openings oval.

Forelimbs short (LAHL/SVL 0.49), strongly hypertrophied; relative finger length III > IV > I > II, first finger slightly longer than second; finger tips rounded, not dilated; webbing between fingers absent; subarticular tubercles distinct, rounded, formula 1, 1, 2, 2, distal ones on fingers III and IV small, others large and prominent; supernumerary tubercles absent; inner metacarpal tubercle large, oval; outer metacarpal tubercle small, slightly elongated; dorsal surfaces of inner metacarpal tubercle and finger I, inner surface of finger II, and inner surface of base of finger III with keratinized nuptial spines.

Hindlimbs long (TiL/SVL 0.52, TL/SVL 0.48, TFL/SVL 0.74), stout; relative toe length IV > III > V > II > I; tips of toes rounded, not dilated; webbing between toes developed, all reached toe tips, obviously incurved between toes; lateral fringe on outer sides of toes I and V present; subarticular tubercles distinct and prominent, formula 1, 1, 2, 3, 2, distal ones on fingers III and IV small and rounded, others large and oval; supernumerary tubercles absent; inner metatarsal tubercles elongated; outer metatarsal tubercle absent; tarsal fold present.

Dorsal skin of head and body rough with many rounded, oval, and elongated warty tubercles; dorsal skin of forelimbs relatively smooth with some small tubercles; dorsal skin of hindlimbs rough with many rounded and oval warty tubercles; ventral skin smooth; some small keratinized spines on ventral surface of head, chest with two oval clusters of large keratinized spines, far apart from each other.

##### Coloration of holotype in life.

Dorsal surface almost uniformly brown, an indistinct dark transversal stripe between eyes, dark transversal bands on limbs nearly invisible; ventral surface of head gray mixed with some light yellow; ventral surface of body and limbs orange yellow, some light gray spots on abdomen; upper iris dark brown, lower iris brownish gray.

##### Variation.

The mensural characteristics of the paratypes are similar to those of the holotype except for the difference in body size (Table 4). The skin texture of the paratypes is similar to that of the holotype with small variations, in that the warty tubercles on dorsum are elongated in some individuals while those are oval or rounded in some individuals. The coloration of the paratypes is also similar to that of the holotype, which all have brownish dorsal surface and orange yellow ventral surface, except for that the dorsal color is paler in some individuals and the ventral surface of head is yellowish white in some individuals. In addition, the forelimbs are not hypertrophied, there are no nuptial spines on chest, inner metacarpal tubercle, and fingers II–III, but only some small keratinized spines on finger I in females.

**Table 4.** Measurements (in mm) of the type specimens of *Nanorana wenshanensis* sp. nov. For abbreviations, see the Materials and methods section.

	KIZ2015051 Holotype ♂	KIZ2015052 Paratype ♂	KIZ2015011 Paratype ♂	KIZ2015053 Paratype ♀	KIZ2015054 Paratype ♀	KIZ2015055 Paratype ♀	KIZ2015056 Paratype ♀	KIZ2015057 Paratype ♀
SVL	102.3	107.1	83.1	110.0	102.3	97.1	95.4	94.0
HL	38.3	40.5	32.6	40.7	38.8	38.3	37.3	36.7
HW	43.1	46.6	36.1	47.3	43.1	42.9	40.2	38.8
SL	16.1	17.6	14.2	16.0	15.8	15.6	15.3	14.7
ED	9.4	10.4	8.6	9.4	9.3	9.6	8.3	9.4
UEW	8.7	9.0	6.3	7.9	8.2	8.7	7.3	8.0
IOS	5.3	6.8	5.4	5.5	6.1	5.2	5.6	5.1
INS	9.4	9.2	7.7	9.6	8.6	8.9	8.6	8.7
NSD	10.0	10.1	8.7	9.8	9.3	9.2	9.0	9.1
NED	6.1	6.7	5.6	6.8	6.6	6.3	6.0	5.7
AED	16.7	17.4	13.3	16.7	16.4	16.1	14.5	14.6
PED	25.1	27.4	21.8	25.6	25.2	25.1	22.8	23.6
TD	5.3	6.4	4.5	5.8	5.5	4.9	5.1	5.2
TED	6.6	6.6	4.7	7.3	6.9	6.4	5.8	5.8
LAHL	49.7	56.1	41.8	52.9	49.9	45.9	45.4	44.9
HaL	29.5	32.5	25.1	30.8	29.7	26.4	26.5	26.9
IPTL	8.9	10.9	7.3	5.8	6.0	5.3	4.6	5.0
IPTW	5.2	6.5	4.4	4.2	3.7	3.3	3.4	3.3
OPTL	4.2	4.6	2.0	4.3	5.5	3.4	3.0	3.1
OPTW	1.5	1.4	1.5	1.9	1.6	1.1	1.1	1.1
TiL	52.7	56.4	44.8	56.7	54.4	50.3	49.8	50.6
TL	49.3	50.7	42.5	53.3	49.6	45.8	47.0	46.8
TW	21.3	20.5	17.1	21.9	19.8	18.7	18.9	18.6
TFL	75.9	81.7	66.1	82.0	78.2	71.2	71.5	73.1
FL	51.3	57.3	45.0	57.4	52.4	48.8	48.0	50.0
IML	7.4	7.9	6.6	8.0	7.2	7.1	7.2	6.4
IMW	2.8	2.5	1.9	2.5	2.9	2.3	2.4	2.1

##### Etymology.

The name refers to Wenshan City, the locality where the new species was found. We propose “Wenshan Spiny Frog” for the common English name and “文山棘蛙” for the common Chinese name of the new specie.

##### Distribution.

This species is currently known to be only distributed in Wenshan City, Wenshan Prefecture, Yunnan Province, China. It is speculated that it may also be distributed in other areas of Wenshan Prefecture at similar altitudes.

##### Natural history notes.

The specimens of *Nanorana wenshanensis* sp. nov. were collected in mountain streams at night. They float in the water near the shore or sit on the shore near the water at night. No courtship calls were heard, and no eggs or tadpoles were found. Their breeding habits remain unknown.

##### Comparisons.

*Nanorana wenshanensis* sp. nov. differs from clade A by having an indistinct tympanum (vs a distinct tympanum); large round, elliptic, and elongate warty tubercles on dorsal surface (vs small round and elliptic or elongate warty tubercles on dorsal surface); warty tubercles on interorbital space (vs no warty tubercles on interorbital space); and an orange yellow ventral surface (vs a white, grayish white, yellowish white, or light yellow ventral surface).

*Nanorana wenshanensis* sp. nov. differs from clade B by having relatively sparser warty tubercles on dorsal surface (vs relatively denser warty tubercles on dorsal surface); lateral surface of head being smooth with no or a few relatively indistinct tubercles (vs lateral surface of head being rough with some relatively distinct tubercles); and spines on ventral surface of head being restricted on each side of lower jaw in adult males (vs spines on ventral surface of head being distributed along entire lower jaw margin or nearly covering entire ventral surface of head in adult males).

*Nanorana wenshanensis* sp. nov. differs from clade C by having large round, elliptic, and elongate warty tubercles on dorsal surface (vs small round and elliptic or elongate warty tubercles on dorsal surface); warty tubercles on interorbital space (vs no warty tubercles on interorbital space); and an orange yellow ventral surface (vs a white, grayish white, flesh colored, or yellowish white ventral surface).

#### Nanorana zhaotongensis sp. nov.

Taxon classificationAnimaliaAnuraDicroglossidae

05C55DAD-C9EC-5C2B-9111-72AB3E8585E3

https://zoobank.org/C0148E2F-8B17-4442-8202-21A46CCEC04C

Figs 6, 7, 8

##### Type material.

***Holotype***. • KIZ2015115, adult male, collected on 25 September 2015 by Dingqi Rao from Tianxing Town, Daguan County, Zhaotong City, Yunnan Province, China (27°52'44"N, 104°5'5"E, 1920 m asl). ***Paratypes***. • KIZ2015114 and KIZ2015097, two adult males; • KIZ2015098 and KIZ2015116–KIZ2015118, four adult females, all the same collection information as the holotype.

**Figure 6.**
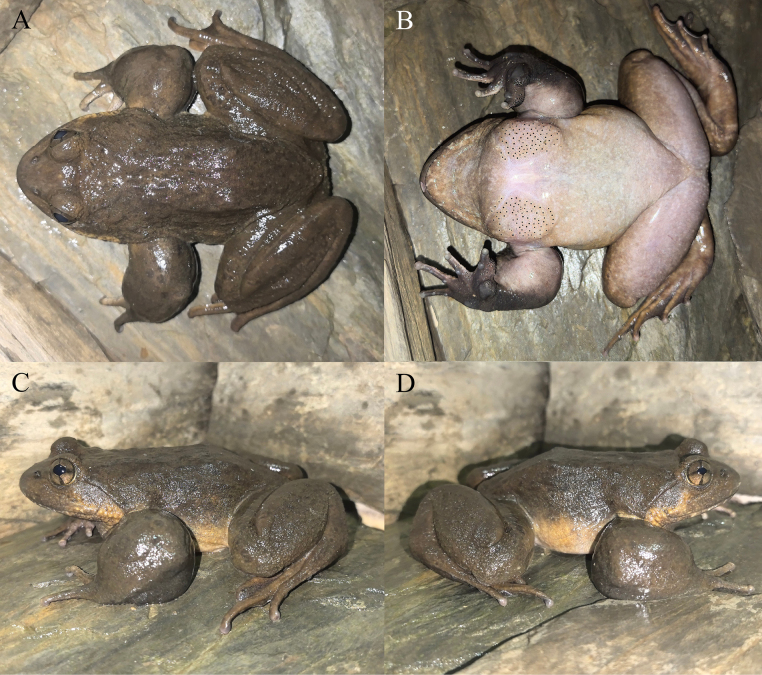
The holotype (KIZ2015115) of *Nanorana zhaotongensis* sp. nov. in life. **A**. Dorsal view; **B**. Ventral view; **C**. Left view; **D**. Right view.

**Figure 7.**
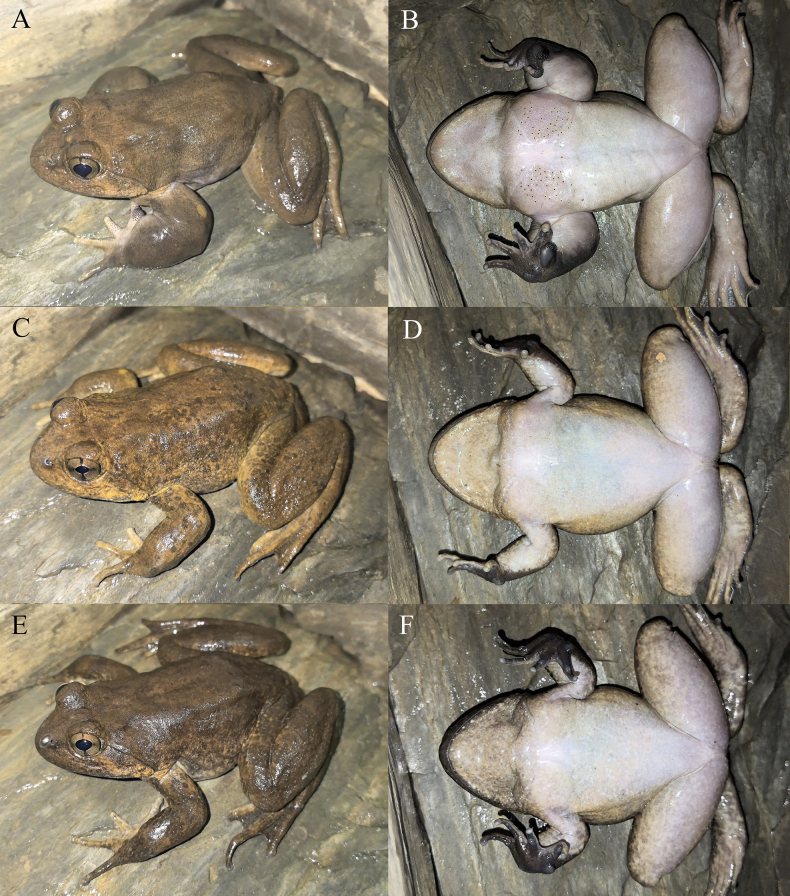
The paratypes of *Nanorana zhaotongensis* sp. nov. in life. **A, B**. The male KIZ2015114; **C, D**. The female KIZ2015116; **E, F**. The female KIZ2015117.

**Figure 8.**
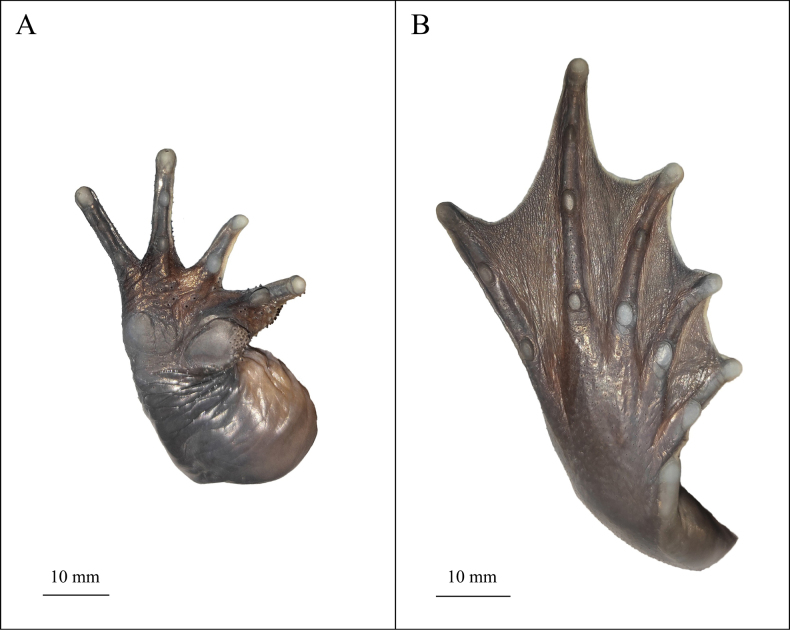
Close-up views of the hand and foot of the holotype (KIZ2015115) of *Nanorana zhaotongensis* sp. nov. in preservative. **A**. Volar view of the right hand; **B**. Plantar view of the right foot.

##### Diagnosis.

Large sized spiny frog, SVL 106–114 mm in adult males and 98–103 mm in adult females; dorsal skin relatively smooth with some flat tubercles; tympanum relatively distinct; finger I slightly longer than finger II; toe III slightly longer than toe V; toes fully webbed but obviously incurved between toes; tarsal fold present; dorsal surface brownish yellow to dark brown; no dark transversal bands on limbs; forelimbs strongly hypertrophied, keratinized spines present on chest, inner metacarpal tubercle, and fingers I to III, and internal vocal sac present in adult males.

##### Description of holotype.

Adult male, habitus stout, SVL 114.3 mm; head broader than long (HW/HL 1.16); snout relatively short (SL/HL 0.43), blunt and rounded in dorsal and lateral views; canthus rostralis obtuse; tympanum relatively distinct; supratympanic fold distinct, from posterior corner of eye to before forelimb insertion; eye moderate (ED/HL 0.24), pupil almost rhombic; choanae oval; vomerine teeth distinct; tongue pear shaped, notched posteriorly; vocal sac openings oval.

Forelimbs short (LAHL/SVL 0.50), strongly hypertrophied; relative finger length III > IV > I > II, first finger slightly longer than second; finger tips rounded, not dilated; webbing between fingers absent; subarticular tubercles distinct and prominent, rounded, formula 1, 1, 2, 2; supernumerary tubercles absent; inner metacarpal tubercle large, oval; outer metacarpal tubercle small, slightly elongated; dorsal surfaces of inner metacarpal tubercle and fingers I and II and inner surface of finger III with keratinized nuptial spines.

Hindlimbs long (TiL/SVL 0.55, TL/SVL 0.52, TFL/SVL 0.77), stout; relative toe length IV > III > V > II > I; tips of toes rounded, not dilated; webbing between toes developed, all reached toe tips, obviously incurved between toes; lateral fringe on outer sides of toes I and V present; subarticular tubercles distinct and prominent, oval, formula 1, 1, 2, 3, 2; supernumerary tubercles absent; inner metatarsal tubercles elongated; outer metatarsal tubercle absent; tarsal fold present.

Dorsal skin of head and body relatively smooth with some oval and elongated flat tubercles; dorsal skin of forelimbs smooth; dorsal skin of hindlimbs relatively smooth with some small rounded tubercles; ventral skin smooth; some small keratinized spines on sides of ventral surface of head, chest with two oval clusters of keratinized spines far apart from each other.

##### Coloration of holotype in life.

Dorsal surface almost uniformly yellowish brown, some small black spots on dorsum and dorsal surface of limbs, no visible dark transversal bands on limbs; ventral surface of head light brown mixed with some yellowish white; ventral surface of body and limbs brownish white; iris brownish gray with a cross shaped black pattern through pupil.

##### Variation.

The mensural characteristics of the paratypes are similar to those of the holotype except for the difference in body size (Table 5). The skin texture of the paratypes is similar to that of the holotype with small variations, in that the flat tubercles on dorsum are relatively distinct in some individuals while those are almost invisible in some individuals. The coloration of the paratypes is also similar to that of the holotype, which all have yellowish brown dorsal surface and whitish ventral surface, except for that the dorsal color is paler in some individuals. In addition, the forelimbs are not hypertrophied, there are no nuptial spines on chest, inner metacarpal tubercle, and fingers in females.

**Table 5.** Measurements (in mm) of the type specimens of *Nanorana zhaotongensis* sp. nov. For abbreviations, see the Material and methods section.

	KIZ2015115 Holotype ♂	KIZ2015114 Paratype ♂	KIZ2015097 Paratype ♂	KIZ2015098 Paratype ♀	KIZ2015116 Paratype ♀	KIZ2015117 Paratype ♀	KIZ2015118 Paratype ♀
SVL	114.3	110.0	106.6	101.1	102.6	97.7	100.9
HL	41.1	41.6	40.3	39.3	37.7	36.2	37.1
HW	47.5	45.6	44.7	42.9	42.4	41.4	41.0
SL	17.5	16.5	16.3	15.5	14.2	14.9	14.2
ED	9.9	10.2	10.2	9.9	11.0	9.4	9.2
UEW	9.3	9.0	9.1	8.4	8.8	8.8	7.7
IOS	7.6	6.4	6.6	6.3	6.4	5.8	6.5
INS	10.8	9.9	10.6	10.5	9.6	9.8	10.0
NSD	9.8	10.0	10.2	8.7	8.6	9.5	8.7
NED	6.5	5.9	6.2	5.8	5.6	5.2	5.4
AED	16.7	16.2	16.5	16.4	16.1	16.0	16.2
PED	27.8	27.2	26.0	25.0	26.1	25.2	25.0
TD	5.1	4.9	4.7	4.7	4.8	4.0	5.1
TED	6.1	6.3	6.3	5.6	5.7	5.7	5.2
LAHL	57.0	52.0	53.5	48.6	48.4	47.7	48.2
HaL	32.2	27.3	28.2	25.7	24.7	26.2	25.4
IPTL	10.6	9.4	9.5	6.3	6.6	5.4	6.3
IPTW	7.6	7.2	6.7	4.2	4.6	4.2	4.3
OPTL	6.8	6.5	6.0	5.4	5.2	5.1	5.4
OPTW	3.6	2.7	2.4	2.4	2.7	3.0	2.4
TiL	63.4	61.0	57.6	57.1	56.0	54.9	57.1
TL	59.8	56.9	56.6	55.0	53.8	53.9	54.1
TW	22.2	21.3	20.3	19.2	18.9	19.5	18.1
TFL	87.6	81.8	81.2	78.5	76.6	79.8	78.2
FL	61.6	56.8	55.9	53.9	52.9	55.3	54.3
IML	8.8	8.8	7.7	7.5	7.9	7.1	7.4
IMW	3.0	2.7	2.4	2.3	2.6	2.3	2.5

##### Etymology.

The name refers to Zhaotong City, the locality where the new species was found. We propose Zhaotong Spiny Frog for the common English name and 昭通棘蛙 for the common Chinese name of the new specie.

##### Distribution.

This species is currently known to be only distributed in Daguan County, Zhaotong City, Yunnan Province, China. It is speculated that it may also be distributed in other areas of Zhaotong City at similar altitudes.

##### Natural history notes.

The specimens of *Nanorana zhaotongensis* sp. nov. were collected in small rivers. They float in the water near the shore or sit on the rocks in the water at night. No courtship calls were heard, and no eggs or tadpoles were found. Their breeding habits remain unknown.

##### Comparisons.

*Nanorana zhaotongensis* sp. nov. differs from clade A by having relatively smooth skin, dorsal surface of body with no or some flat tubercles, lateral surface of body with some flat tubercles, and dorsal surface of hindlimbs with some indistinct small tubercles (vs relatively rough dorsal skin, dorsal surface of body with some small round and elliptic or elongate warty tubercles, lateral surface of body with some small round or elliptic warty tubercles, and dorsal surface of hindlimbs with some distinct small tubercles).

*Nanorana zhaotongensis* sp. nov. differs from clade B by having a distinct tympanum (vs an indistinct tympanum); much smoother skin, dorsal surface with sparse flat tubercles (vs much rougher skin, dorsal surface with denser warty tubercles); no warty tubercles on interorbital space (vs warty tubercles on interorbital space); lateral surface of head being smooth with no or a few relatively indistinct tubercles (vs lateral surface of head being rough with some relatively distinct tubercles); and no spines on ventral surface of head or spines on ventral surface of head being restricted on each side of lower jaw in adult males (vs spines on ventral surface of head being distributed along entire lower jaw margin or nearly covering entire ventral surface of head in adult males).

*Nanorana zhaotongensis* sp. nov. differs from clade C by having a distinct tympanum (vs an indistinct tympanum) and relatively smooth skin, dorsal surface of body with no or some flat tubercles, lateral surface of body with some flat tubercles, and dorsal surface of hindlimbs with some indistinct small tubercles (vs relatively rough dorsal skin, dorsal surface of body with some small elliptic or elongate warty tubercles, lateral surface of body with some small round and elliptic or elongate warty tubercles, and dorsal surface of hindlimbs with some distinct small tubercles).

*Nanorana zhaotongensis* sp. nov. differs from *Nanorana wenshanensis* sp. nov. by having a distinct tympanum (vs an indistinct tympanum); much smoother skin, dorsal surface with some flat tubercles (vs much rougher skin, dorsal surface with many warty tubercles), no warty tubercles on interorbital space (vs warty tubercles on interorbital space), and a white or flesh colored ventral surface (vs an orange yellow ventral surface).

## Discussion

Huang et al. (2016) clarified the taxonomic history of *Rana phrynoides* and *R. yunnanensis* and considered that Dongchuan District of Kunming City is the type locality of *R. phrynoides* and Husa Township of Longchuan County is the type locality of *R. yunnanensis*. Since the syntypes of *R. yunnanensis* had been lost, Dubois (1987) designated a neotype (BMNH 1947.2.3.76) for *R. yunnanensis*. However, the neotype (BMNH 1947.2.3.76) is one of the syntypes of *R. phrynoides* which was collected from Dongchuan. In order to keep these two names, Huang et al. (2016) re-designated a neotype (CIBYN09060612) for *R. yunnanensis* which was collected from Husa. Subsequently, the database of Chinese amphibians adopted the viewpoint of Huang et al. (2016) and listed the three species *N. phrynoides*, *N. sichuanensis*, and *N. yunnanensis* (AmphibiaChina 2026). On the contrary, Frost (2026) considered that the replacing of the neotype by Huang et al. (2016) is ineffective as it does not comply with the International Commission on Zoological Nomenclature Code of nomenclature (ICZN 1999). Since the specimen BMNH 1947.2.3.76 already bears the two names *R. phrynoides* and *R. yunnanensis*, and *R. yunnanensis* appeared earlier than *R. phrynoides*, therefore, Frost (2026) applied the name *R. yunnanensis* to the species to which the specimen BMNH 1947.2.3.76 belongs and resurrected the name *R. bourreti* to represent the species previously referred to by *R. yunnanensis*. However, these historical issues are not the aim of this study, which focuses on describing new species. Regardless of the names of the three known species, it does not affect the validity of the two species described in this study. To reduce confusion, we use clades A–C to represent the three known species of the *N. yunnanensis* complex herein.

Genetic distance is usually used to judge whether two organisms are the same species, and the threshold of genetic distance greater than 2% in mitochondrion gene was suggested for recognizing one species from another in most vertebrate taxa (Hebert et al. 2003a, 2003b, 2004). However, there is no exact genetic distance value that can be used as a clear species border in amphibians (Borkin and Litvinchuk 2008). In this study, the minimum genetic distances between species of the *Nanorana yunnanensis* complex were 2.0% and 7.7% in 16S and ND2, respectively. We speculate that the small genetic distance in 16S may be due to the relative conservation of this gene and the short length of many available sequences. Nevertheless, the minimum genetic distances between the two new species and other species of the *N. yunnanensis* complex were all greater than 2%. This confirms the validity of the two new species.

**Figure 9.**
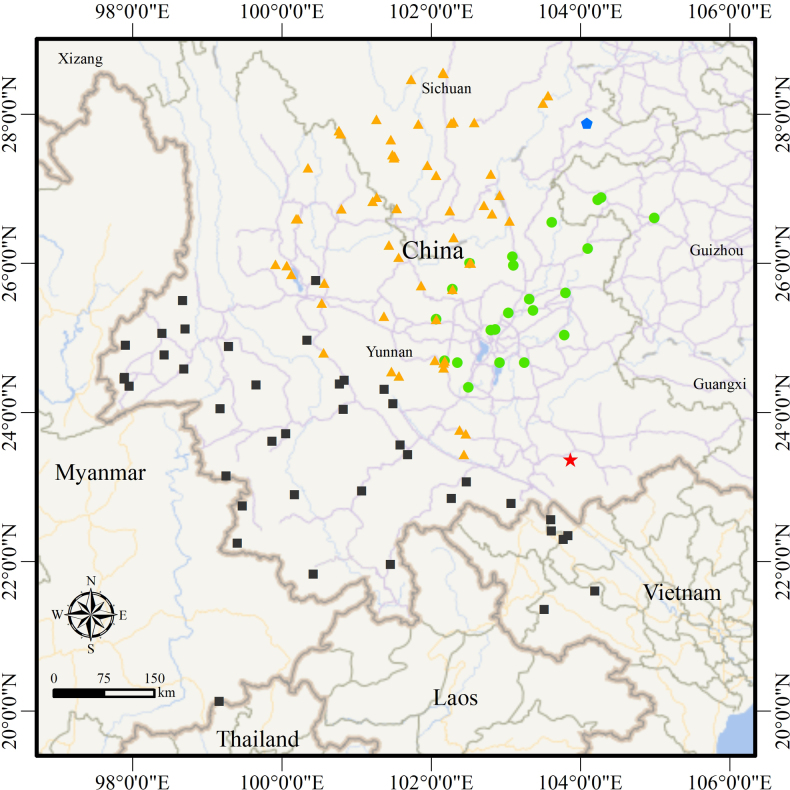
Map showing the general distribution of the *Nanorana yunnanensis* complex. Yellow triangles represent clade A, black squares represent clade B, green dots represent clade C, red star represents *Nanorana wenshanensis* sp. nov., and blue pentagon represents *Nanorana zhaotongensis* sp. nov.

Zhang et al. (2010) and Huang et al. (2016) successively studied the *Nanorana yunnanensis* complex through extensive sampling, both of them included samples from Zhaotong City, but no samples from Wenshan Prefecture. However, their samples from Zhaotong City are limited to Qiaojia County and do not include samples from other areas of Zhaotong City. In this study, we included samples from Daguan County of Zhaotong City and Wenshan Prefecture and found that these samples can be distinguished from other species of the *N. yunnanensis* complex and from each other in both phylogenetic analysis and morphological characteristics. Therefore, we described them as two new species. That is to say, there are two species of the *N. yunnanensis* complex distributed in Zhaotong City, namely Clade A and *Nanorana zhaotongensis* sp. nov. Currently, within Zhaotong City, Clade A is known to be only distributed in Qiaojia County, while *Nanorana zhaotongensis* sp. nov. is known to be only distributed in Daguan County, and no sympatric distribution of these two species has been found (Fig. 9).

**Figure 10.**
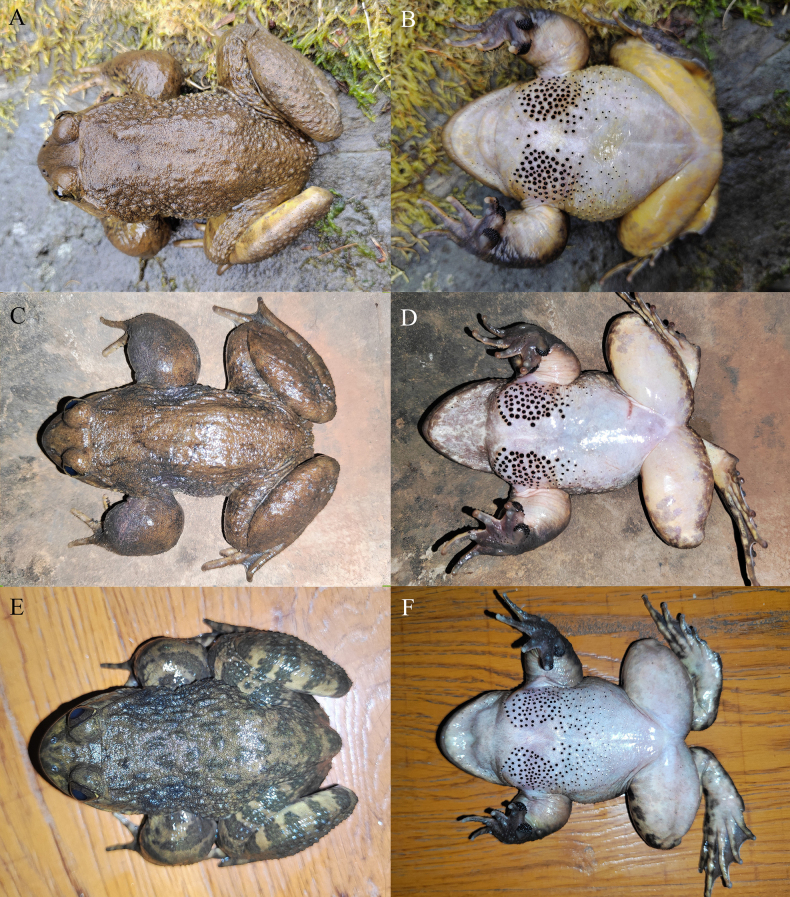
The males of clade A in life. **A, B**. From Ninglang, Lijiang, Yunnan, China; **C, D**. From Yulong, Lijiang, Yunnan, China; **E, F**. From Yinqiao, Dali, Yunnan, China.

**Figure 11.**
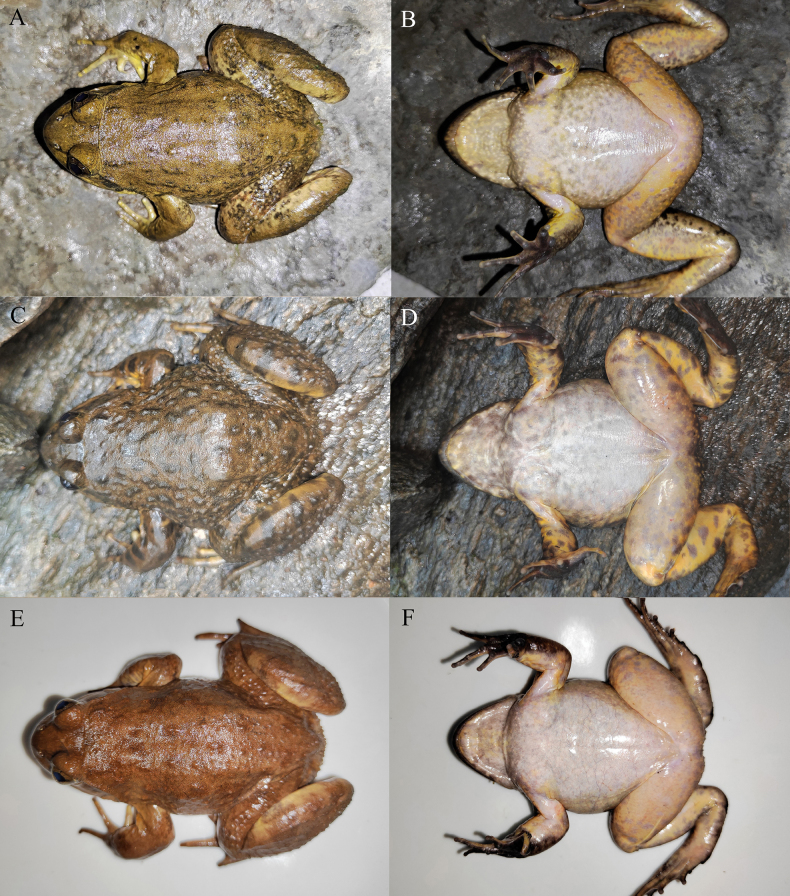
The females of clade A in life. **A, B**. From Ninglang, Lijiang, Yunnan, China; **C, D**. From Dali, Dali, Yunnan, China; **E, F**. From Binchuan, Dali, Yunnan, China.

**Figure 12.**
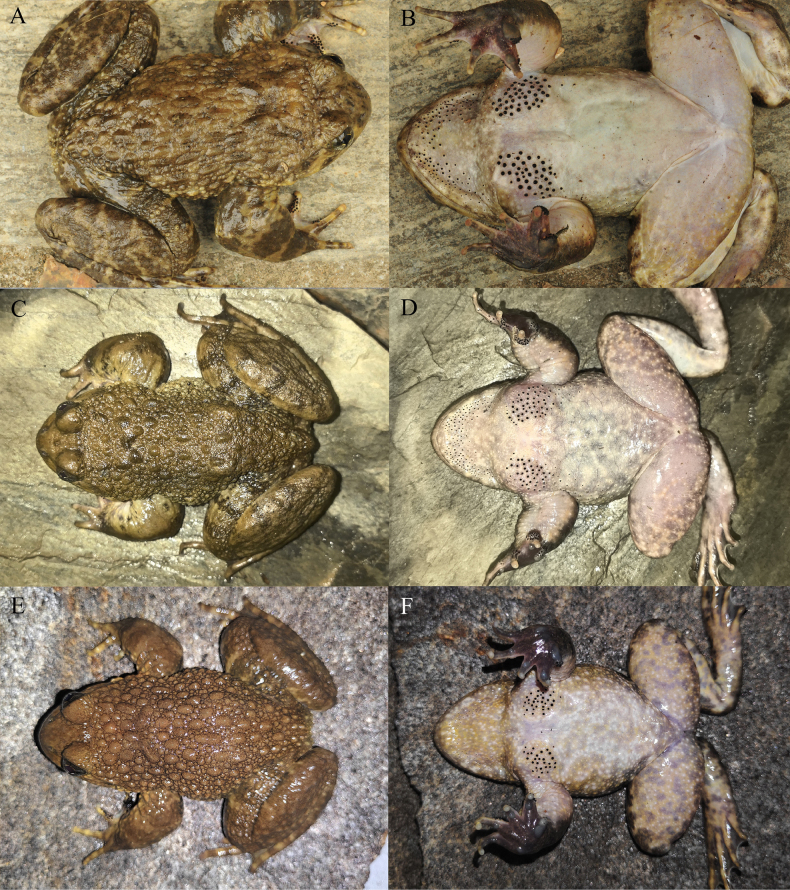
The males of clade B in life. **A, B**. From Longchuan, Dehong, Yunnan, China; **C, D**. From Jingdong, Pu’er, Yunnan, China; **E, F**. From Yuanyang, Honghe, Yunnan, China.

**Figure 13.**
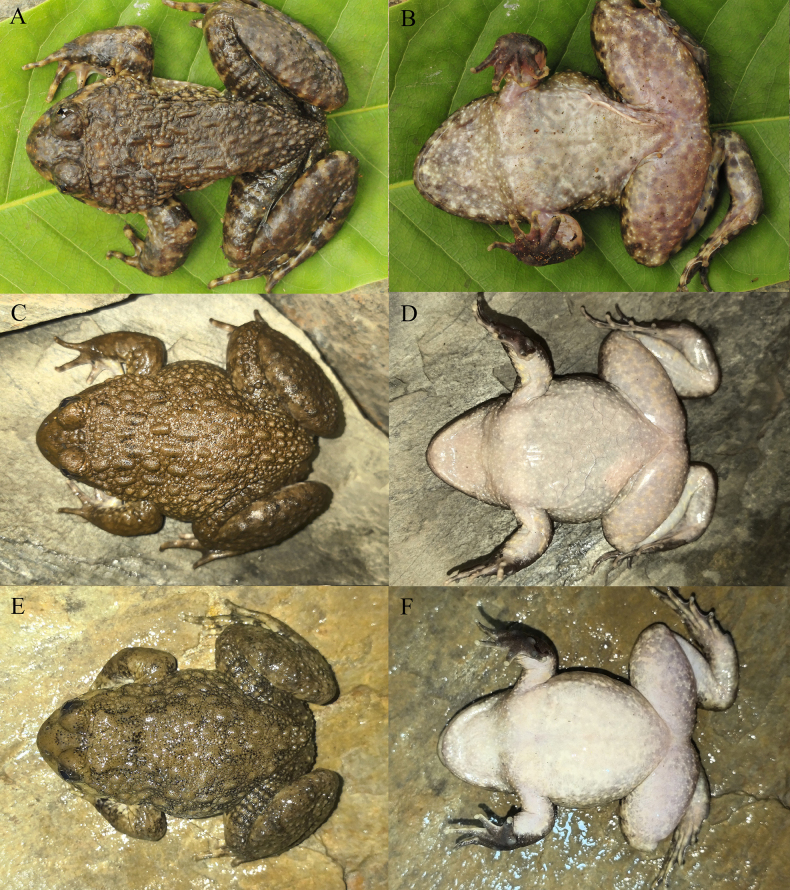
The females of clade B in life. **A, B**. From Longchuan, Dehong, Yunnan, China; **C, D**. From Jingdong, Pu’er, Yunnan, China; **E, F**. From Yuanyang, Honghe, Yunnan, China.

**Figure 14.**
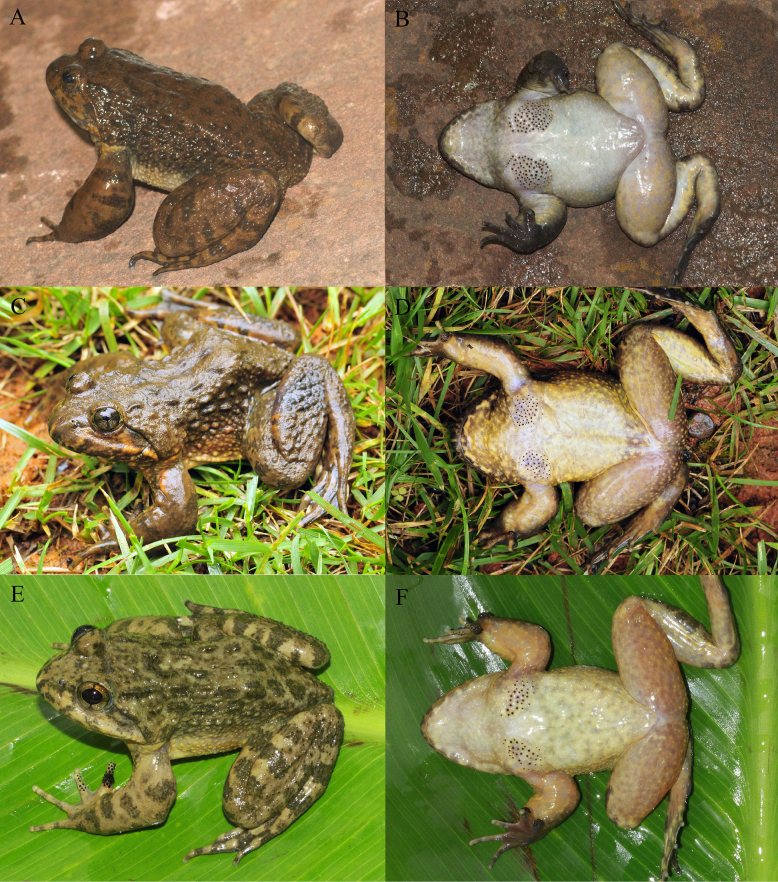
The males of clade C in life. **A, B**. From Guandu, Kunming, Yunnan, China; **C, D**. From Huize, Qujing, Yunnan, China; **E, F**. From Lonjie, Weining, Guizhou, China.

**Figure 15.**
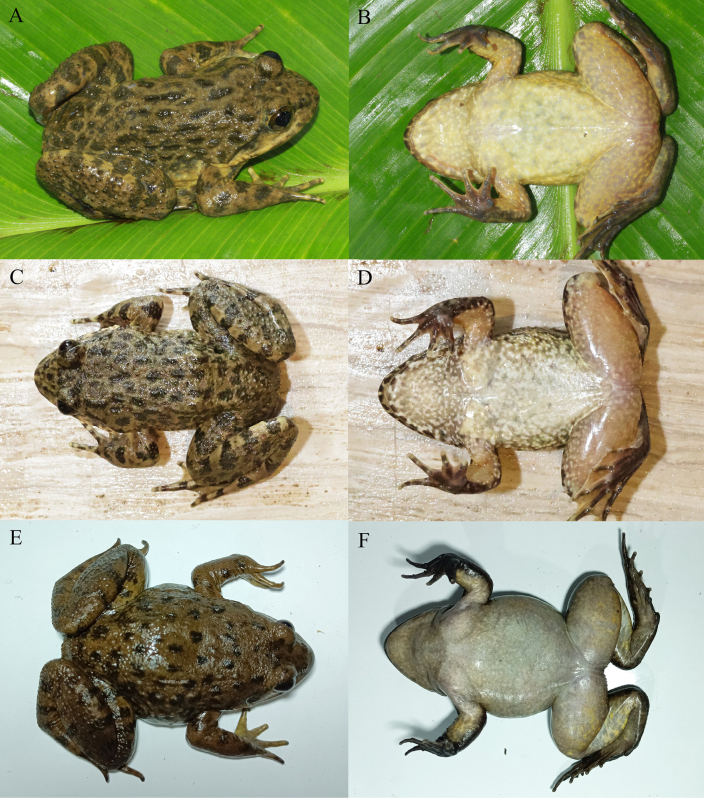
The females of clade C in life. **A, B, C, D**. From Lonjie, Weining, Guizhou, China; **E, F**. From Guandu, Kunming, Yunnan, China.

Huang et al. (2016) distinguish species of the *Nanorana yunnanensis* complex by the characteristics of toe webbing, tympanum, dorsal skin, nuptial spines in males, and ventral coloration. Through our observation of a large number of specimens and photographs of the *N. yunnanensis* complex (Figs 10, 11, 12, 13, 14, 15), we have found that whether the tympanum is distinct and the degree of dorsal skin roughness can indeed be used to distinguish these species. However, whether the toes are fully webbed, whether an inverted “V” pattern composed of warty tubercles present on the back of shoulders, and whether the nuptial spines on chest spread downwards vary significantly within the same species. Therefore, we consider that these characteristics are unstable and cannot be used to distinguish these species. As for the color of the ventral surface, we consider it can be used to distinguish species to a certain extent. The ventral color of clades A, C, and *Nanorana zhaotongensis* sp. nov. is usually white, grayish white, yellowish white, flesh colored, or light yellow, and no orange yellow has been observed. The ventral color of clade B is also usually white, grayish white, yellowish white, flesh colored, or light yellow, but individuals with orange yellow ventral surface are occasionally encountered. According to our observation, the ventral color of *Nanorana wenshanensis* sp. nov. is consistently conspicuous orange yellow, which is stable within this species, and therefore, this characteristic can be used to distinguish it from other species of the *N. yunnanensis* complex except for clade B. In addition, we found that there are some regularities in the characteristic of the spines on the ventral surface of the head in males. The spines on the ventral surface of the head are restricted on each side of the lower jaw in clade A and *Nanorana wenshanensis* sp. nov., there are no spines on the ventral surface of the head or the spines on the ventral surface of the head are restricted on each side of the lower jaw in clade C and *Nanorana zhaotongensis* sp. nov., while the spines on the ventral surface of the head are distributed along the entire lower jaw margin or nearly covering the entire ventral surface of the head in clade B. However, it must be noted that this trait is stable only in large sized, fully mature males, and it is not applicable for comparison between small sized, subadult males.

The specimens of the two new species were all collected ten years ago, and at that time their populations were relatively large. Although these species are somewhat threatened by human capture, their distribution areas are within nature reserves. The current survival status of these two species is unknown, but we speculate that they are not endangered. Future field surveys are needed to clarify their conservation status.

### Key to the species of the *Nanorana yunnanensis* complex

**Table:** 

1	Warty tubercles present on interorbital space	**2**
–	Warty tubercles absent on interorbital space	**3**
2	Dorsal skin with many dense warty tubercles, skin on lateral head relatively rough	**clade B**
–	Dorsal skin with some sparse warty tubercles, skin on lateral head relatively smooth	***Nanorana wenshanensis* sp. nov**.
3	Dorsal skin rough with warty tubercle	**4**
–	Dorsal skin smooth with flat tubercle	***Nanorana zhaotongensis* sp. nov**.
4	Tympanum distinct, upper lip color usually uniform	**clade A**
–	Tympanum indistinct, upper lip color usually mottled	**clade C**

## Supplementary Material

XML Treatment for Nanorana wenshanensis

XML Treatment for Nanorana zhaotongensis

## Additional information

### Artificial Intelligence (AI) Use

No use of AI was reported.

### Author ORCIDs

Shuo Liu https://orcid.org/0000-0001-7825-3006

Mian Hou https://orcid.org/0000-0002-3322-6570

JiShan Wang https://orcid.org/0000-0003-4660-163X

Dingqi Rao https://orcid.org/0000-0003-2680-1503

### Data availability

All of the data that support the findings of this study are available in the main text or Supplementary Information.

## Appendix 1

**Specimens examined**

Clade A (*n* = 45): KIZ 90146–90147, 910149, 91060486–91060487, females, KIZ 910148, 910450, 0074, males, Dayao, Chuxiong, Yunnan, China; KIZ 9100094, male, Gucheng, Lijiang, Yunnan, China; KIZ2025079, male, KIZ2025080, female, Sanba, Xianggelila, Yunnan, China; KIZ2025077–2025078, 2025081–2025082, males, KIZ2025176, female, Ninglang, Lijiang, Yunnan, China; KIZ2025087, female, Binchuan, Dali, Yunnan, China; KIZ2025083–2025084, 2025179–2025180, males, Baisha, Lijiang, Yunnan, China; KIZ2025085–2025086, 2025182, 2025184–2025186, males, KIZ2025181, 2025183, 2025187–2025190, females, Xizhou, Dali, Yunnan, China; KIZ2025092, male, KIZ2025191, female, Yinqiao, Dali, Yunnan, China; KIZ2025091, 2025192–2025193, males, KIZ2025194–2025195, females, Dali, Dali, Yunnan, China; KIZ2025093, 2025196–2025197, males, KIZ2025198–2025199, females, Xiaguan, Dali, Yunnan, China.

Clade B (*n* = 61): KIZ 035315–035319, females, KIZ 035320, male, Yingjiang, Dehong, Yunnan, China; KIZ 74II0144–74II0145, 74II0154–74II0157, 025198, males, KIZ 74II0146, 74II0158–74II0159, 025199, 025201, 025203, females, Longchuan, Dehong, Yunnan, China; KIZ 820697, 040200, males, KIZ 820730–820731, 035172, females, Tengchong, Baoshan, Yunnan, China; KIZ 035690, 035695–035696, males, KIZ 035679–035680, 035691, 035693, 035697, Longling, Baoahan, Yunnan, China; KIZ 035174, male, KIZ 035175, female, Longyang, Baoshan, Yunnan, China; KIZ 75II0213, 75II0215, 75II0231–75II0232, 75II0282–75II0283, 75II0285, 75II0473, 75II0475, 75II1095, males, KIZ 75II0116, 75II0120, 75II0229, 75II0230, 75II0233, 75II0242, 75II0281, 75II0284, 75II0307, females, Jingdong, Pu’er, Yunnan, China; KIZ 85I0190, 85I0227, 85I0254, 85I219, males, KIZ 85I0221–85I0222, 85I0225, 85I0257, females, Lüchun, Honghe, Yunnan, China.

Clade C (*n* = 29): KIZ 019631, male, Anning, Kunming, Yunnan, China; KIZ 019458, female, Songming, Kunming, Yunnan, China; KIZ 020999, female, Luliang, Qujing, Yunnan, China; KIZ 019791–019792, 019785–019789, 019795–019799, 019802, 019805, 019807–019808, 019810–019811, males, KIZ 019749, 019788, 019793, 019800–019801, 0198043–019804, 019806, females, Bijie, Weining, Guizhou, China; KIZ 021710, female, Yiliang, Kunming, Yunnan, China.

## Supplementary materials

Supplementary material 1 Measurements of examined specimens Data type xlsx Explanation note Detailed measurements of each examined specimen of the *Nanorana yunnanensis* complex. https://binary.pensoft.net/file/1582276This dataset is made available under the Open Database License (http://opendatacommons.org/licenses/odbl/1.0/). The Open Database License (ODbL) is a license agreement intended to allow users to freely share, modify, and use this Dataset while maintaining this same freedom for others, provided that the original source and author(s) are credited. Shuo Liu, Chao Bu, Mian Hou, Jing Chen, Yanfei Feng, Lue Ma, JiShan Wang, Dingqi Rao
